# Toward Using Twitter Data to Monitor COVID-19 Vaccine Safety in Pregnancy: Proof-of-Concept Study of Cohort Identification

**DOI:** 10.2196/33792

**Published:** 2022-01-06

**Authors:** Ari Z Klein, Karen O'Connor, Graciela Gonzalez-Hernandez

**Affiliations:** 1 Department of Biostatistics, Epidemiology, and Informatics Perelman School of Medicine University of Pennsylvania Philadelphia, PA United States

**Keywords:** natural language processing, social media, COVID-19, data mining, COVID-19 vaccine, pregnancy outcomes

## Abstract

**Background:**

COVID-19 during pregnancy is associated with an increased risk of maternal death, intensive care unit admission, and preterm birth; however, many people who are pregnant refuse to receive COVID-19 vaccination because of a lack of safety data.

**Objective:**

The objective of this preliminary study was to assess whether Twitter data could be used to identify a cohort for epidemiologic studies of COVID-19 vaccination in pregnancy. Specifically, we examined whether it is possible to identify users who have reported (1) that they received COVID-19 vaccination during pregnancy or the periconception period, and (2) their pregnancy outcomes.

**Methods:**

We developed regular expressions to search for reports of COVID-19 vaccination in a large collection of tweets posted through the beginning of July 2021 by users who have announced their pregnancy on Twitter. To help determine if users were vaccinated during pregnancy, we drew upon a natural language processing (NLP) tool that estimates the timeframe of the prenatal period. For users who posted tweets with a timestamp indicating they were vaccinated during pregnancy, we drew upon additional NLP tools to help identify tweets that reported their pregnancy outcomes.

**Results:**

We manually verified the content of tweets detected automatically, identifying 150 users who reported on Twitter that they received at least one dose of COVID-19 vaccination during pregnancy or the periconception period. We manually verified at least one reported outcome for 45 of the 60 (75%) completed pregnancies.

**Conclusions:**

Given the limited availability of data on COVID-19 vaccine safety in pregnancy, Twitter can be a complementary resource for potentially increasing the acceptance of COVID-19 vaccination in pregnant populations. The results of this preliminary study justify the development of scalable methods to identify a larger cohort for epidemiologic studies.

## Introduction

COVID-19 during pregnancy is associated with an increased risk of maternal death, intensive care unit admission, and preterm birth [1]; however, in the United States, uptake of COVID-19 vaccination during pregnancy is low [2]. Surveys indicated that the most common reason for refusing COVID-19 vaccination during pregnancy was a lack of safety data [3], which are limited because people who were pregnant were excluded from preauthorization clinical trials. The Centers for Disease Control and Prevention (CDC) recently released the first US data on COVID-19 vaccine safety in pregnancy, based on postvaccination health information reported by participants voluntarily enrolled in V-safe [4]. According to the CDC, although the preliminary data do not indicate any obvious safety signals, continued monitoring is needed, especially in early pregnancy and the periconception period (within 30 days before the last menstrual period). The CDC suggests that additional evidence of COVID-19 vaccine safety in pregnancy is critical for increasing the acceptance of COVID-19 vaccination in pregnant populations [2].

In the United States, 42% of people aged 18-29 years and 27% of people aged 30-49 years use Twitter [5]. Our prior work [6] demonstrated that Twitter data can be used to assess outcomes associated with medication exposure during pregnancy. Therefore, we hypothesized that Twitter could also be a source of data for assessing outcomes associated with COVID-19 vaccination received during pregnancy. Although user-generated Twitter data may be subject to potential limitations similar to those that the CDC has discussed regarding their participant-reported data [4] (eg, selection bias, reporting bias, misreporting, small sample size, limited information on other risk factors), the current availability of other sources of data is very limited. The objective of this preliminary study was to assess whether Twitter data could be used to identify a cohort for epidemiologic studies of COVID-19 vaccination in pregnancy. In particular, we explored whether it is possible to identify users who have reported (1) that they received COVID-19 vaccination during pregnancy or the periconception period, and (2) their pregnancy outcomes.

## Methods

The Institutional Review Board of the University of Pennsylvania reviewed this study and deemed it exempt from human subjects research under Category 4 of Paragraph b of the US Code of Federal Regulations Title 45 Section 46.101 for publicly available data sources (45 CFR §46.101(b)(4)).

To facilitate a preliminary assessment of self-reports of COVID-19 vaccination on Twitter, we developed 6 handwritten, high-precision regular expressions designed to match tweets mentioning that the user received at least one dose of COVID-19 vaccination (Multimedia Appendix 1). In prior work [7], we developed an automated natural language processing (NLP) pipeline that detects tweets from the Twitter streaming application programming interface that announce a user’s pregnancy, and then collects all of their publicly available tweets on an ongoing basis. We deployed the 6 regular expressions on the collection of these users’ tweets that were posted through to the beginning of July 2021. To help determine if users were vaccinated during pregnancy (or the periconception period), we manually compared the timestamp of the tweets that matched the regular expressions with the timeframe of their prenatal period. To help estimate the timeframe of the users’ prenatal period, we drew upon an automated NLP tool, developed in our prior work [8], that uses a rule-based approach to search tweets for reports of the baby’s gestational age, due date, or date of birth, and extracts an estimate of the beginning and end dates of pregnancy based on the specific information in the tweet.

For users who posted tweets with a timestamp indicating they were vaccinated during pregnancy or the periconception period, we drew upon additional automated NLP tools, developed in our prior work [9-11], that use supervised classification to search tweets for reports of adverse pregnancy outcomes, including miscarriage, stillbirth, preterm birth, low birth weight, birth defects, and neonatal intensive care unit admission. To reduce the potential reporting bias in assuming that the lack of tweets self-reporting an adverse pregnancy outcome represents the lack of an adverse outcome, we also deployed an automated NLP tool, developed in our prior work [12], to search users’ tweets for reports that the baby was born at a gestational age of at least 37 weeks (ie, that the user is at least 37 weeks pregnant, or that the due date is in 3 weeks or less) and a weight of at least 5 pounds and 8 ounces. A gestational age of at least 37 weeks indicates the lack of miscarriage or preterm birth. A birth weight of at least 5 pounds and 8 ounces indicates the lack of low birth weight or, as a report of live birth, miscarriage, or stillbirth. If we did not automatically detect a tweet explicitly reporting a gestational age of at least 37 weeks, we manually analyzed tweets posted during this time for evidence that the user was still pregnant.

## Results

We manually verified the content of tweets detected automatically, identifying 150 users who reported on Twitter that they received at least one dose of COVID-19 vaccination during pregnancy or the periconception period. Table 1 presents examples of tweets that we used to identify these 150 users. For example, user 1 reported being 16 weeks pregnant on June 15, 2021, and therefore our automated tool [8] estimated that pregnancy began on February 23, 2021. User 1 reported receiving COVID-19 vaccination on March 24, 2021, which is approximately 1 month into the pregnancy. User 2 reported being 13 weeks pregnant on June 21, 2021, and our automated tool [8] estimated that the pregnancy began on March 22, 2021. User 2 reported receiving COVID-19 vaccination on March 6, 2021, which corresponds to the periconception period. The tweets in Table 1 also show that some users reported the vaccine manufacturer (eg, “#PfizerVaccine”) or dose number (eg, “second vaccine”), which can help distinguish mRNA vaccines from other types. Based on our estimates of the prenatal period for these 150 users, 90 (60.0%) of their pregnancies may have been ongoing. We manually verified at least one reported outcome for 45 of the 60 (75%) completed pregnancies. Table 2 presents the outcomes reported by these 45 users.

**Table 1:** Sample tweets indicating that COVID-19 vaccination was received during pregnancy or the periconception period.

Tweets		Timestamp	Pregnancy start	Pregnancy end
**User 1**				
	I am bringing a life into this world and that is pretty darn incredible. #16weekspregnant	June 15, 2021	February 23, 2021	November 30, 2021
	Got my first dose of the COVID vaccine today and feeling so excited and grateful for science	March 24, 2021	February 23, 2021	November 30, 2021
**User 2**				
	I’m awake because I’m 13 weeks pregnant and...well...“morning sickness”	June 21, 2021	March 22, 2021	December 27, 2021
	Got my #PfizerVaccine last night!	March 7, 2021	March 22, 2021	December 27, 2021
**User 3**				
	93 days till my due date	April 17, 2021	November 11, 2020	August 18, 2021
	So I just got my second vaccine. So far I feel fine... I’m praying it stays that way all day	March 1, 2021	November 11, 2020	August 18, 2021
**User 4**				
	I’m 8 months pregnant. My family drove down to Tampa with me just in case baby shows up early	June 9, 2021	October 9, 2020	July 16, 2021
	Happy to report I received the Moderna vaccine today	March 6, 2021	October 9, 2020	July 16, 2021

**Table 2:** Self-reported pregnancy outcomes for Twitter users who received COVID-19 vaccination during pregnancy or the periconception period (N=45).

Self-reported outcome		Outcomes, n (%)^a^	Sample tweet
**Adverse outcomes**			
	Neonatal intensive care unit (NICU)	5 (11)	I made a small human. So that’s pretty cool. Now for a few weeks of NICU time.
	Preterm birth (<37 weeks)	4 (9)	She was born Jan. 11th...3 months early...stayed in the hospital until about 2.5 weeks ago...
	Low birth weight (<5 pounds, 8 ounces)	1 (2)	He weighed 3 lbs 9 ounces @ birth & we didn’t have 1 thing that came close to fitting him.
	Miscarriage	1 (2)	In the last 4 weeks, I’ve had a miscarriage...family death...pet death...my car broke down...finals...
	Stillbirth	0 (0)	N/A^b^
	Birth defect	0 (0)	N/A
**Normal outcomes**			
	Term (≥37 weeks)^c^	39 (87)	He made his debut at #37weeks. We got to the hospital by 6:15am, fully dilated by 7:45am, and he was here at 8:22am!
	Normal birth weight (≥5 pounds, 8 ounces)	7 (16)	He arrived via c/section last night at 8:49pm. He was 7 lbs 11 oz. I can’t believe he’s mine!

^a^Multiple outcomes were identified for some pregnancies; therefore, the sum and percentage of the total outcomes are greater than 45 and 100%, respectively.

^b^N/A: not applicable.

^c^Pregnancies were included for which we did not find subsequent tweets explicitly indicating live birth.

## Discussion

### Principal Findings

Our study demonstrates that there are users who report on Twitter that they were vaccinated during pregnancy, including in early pregnancy and the periconception period, and that many of them report their pregnancy outcomes. Therefore, the results of this study justify the development of scalable methods to identify a larger cohort on Twitter for epidemiologic studies of COVID-19 vaccination in pregnancy. The 150 users in this study were identified based on tweets posted through the beginning of July 2021. Since identifying these users, we have redeployed the 6 regular expressions for detecting tweets that self-report COVID-19 vaccination and our NLP tool that estimates the timeframe of the prenatal period [8] on users’ tweets [7] collected through November 2021. Even using the regular expressions alone, we have automatically identified approximately 2000 additional users who posted a matching tweet and have an estimated due date in 2021 or 2022. In future work, we will manually verify their tweets to determine the inclusion of these additional users in our cohort of people who received COVID-19 vaccinated during pregnancy or the periconception period. Therefore, the small size of the initial cohort—150 users—seems to largely reflect the point of time in which this study began, rather than the larger-scale utility of Twitter data.

Our preliminary results suggest that reports of preterm birth and miscarriage are largely unaffected by a potential reporting bias, given that we detected a gestational age of at least 37 weeks for 39 of the 40 (98%) completed pregnancies for which we did not identify a preterm birth or miscarriage. However, reports of low birth weight may be affected by a potential reporting bias, given that we detected a birth weight of at least 5 pounds and 8 ounces for only 7 of the 44 (16%) completed pregnancies for which we did not identify a low birth weight. Given our initial small sample of Twitter users, it is not surprising that we did not detect any reports of birth defects or stillbirth, which have an incidence in the United States of 3% [13] and less than 1% [14], respectively. Nonetheless, our prior work [9-11] demonstrates that users do report these rare outcomes on Twitter. Although a full comparison is beyond the scope of this study, out of the total number of pregnancies with a reported gestational age of at least 20 weeks, the proportion of preterm births reported on Twitter (9.09%) is similar to both the incidence in the United States prior to the COVID-19 pandemic (10.23%) [15] and the proportion reported by V-safe participants (9.4%) [4].

### Conclusions

Given the limited availability of data on COVID-19 vaccine safety in pregnancy, Twitter can be a complementary resource for continued monitoring and potentially increasing the acceptance of COVID-19 vaccination in pregnant populations. Directions for future work include developing methods to detect a larger cohort, and performing an epidemiologic study comparing their pregnancy outcomes to those of users who have announced their pregnancy on Twitter [7] but gave birth prior to the availability of COVID-19 vaccines.

## Appendix

Multimedia Appendix 1 Regular expressions for detecting tweets that self-report COVID-19 vaccination.

## Abbreviations

CDC: Centers for Disease Control and Prevention
NLP: natural language processing
